# Distinct and Shared Endophenotypes of Neural Substrates in Bipolar and Major Depressive Disorders

**DOI:** 10.1371/journal.pone.0168493

**Published:** 2016-12-28

**Authors:** Toshio Matsubara, Koji Matsuo, Kenichiro Harada, Masayuki Nakano, Mami Nakashima, Toshio Watanuki, Kazuteru Egashira, Matakazu Furukawa, Naofumi Matsunaga, Yoshifumi Watanabe

**Affiliations:** 1 Division of Neuropsychiatry, Department of Neuroscience, Yamaguchi University Graduate School of Medicine, Ube, Yamaguchi, Japan; 2 Health Service Center, Yamaguchi University Organization for University Education, Yamaguchi, Yamaguchi, Japan; 3 Katakura Hospital, Ube, Yamaguchi, Japan; 4 Nagato-ichinomiya Hospital, Shimonoseki, Yamaguchi, Japan; 5 Egashira Clinic, Kitakyusyu, Fukuoka, Japan; 6 Department of Radiology, Yamaguchi University Graduate School of Medicine, Ube, Yamaguchi, Japan; Chinese Academy of Sciences, CHINA

## Abstract

Little is known about disorder-specific biomarkers of bipolar disorder (BD) and major depressive disorder (MDD). Our aim was to determine a neural substrate that could be used to distinguish BD from MDD. Our study included a BD group (10 patients with BD, 10 first-degree relatives (FDRs) of individuals with BD), MDD group (17 patients with MDD, 17 FDRs of individuals with MDD), and 27 healthy individuals. Structural and functional brain abnormalities were evaluated by voxel-based morphometry and a trail making test (TMT), respectively. The BD group showed a significant main effect of diagnosis in the gray matter (GM) volume of the anterior cingulate cortex (ACC; *p* = 0.01) and left insula (*p* < 0.01). FDRs of individuals with BD showed significantly smaller left ACC GM volume than healthy subjects (*p* < 0.01), and patients with BD showed significantly smaller ACC (*p* < 0.01) and left insular GM volume (*p* < 0.01) than healthy subjects. The MDD group showed a tendency toward a main effect of diagnosis in the right and left insular GM volume. The BD group showed a significantly inverse correlation between the left insular GM volume and TMT-A scores (*p* < 0.05). Our results suggest that the ACC volume could be a distinct endophenotype of BD, while the insular volume could be a shared BD and MDD endophenotype. Moreover, the insula could be associated with cognitive decline and poor outcome in BD.

## Introduction

Differentiating between a diagnosis of bipolar disorder (BD) and major depressive disorder (MDD) is of clinical importance, as 69% of individuals with BD are misdiagnosed with MDD [1]. These errors cause harmful consequences for patients with BD, such as inappropriate medication prescription, drug-induced switching of the mood phase, and a poor prognosis. Although empirical markers that could allow these two disorders to be distinguished are desired, the biological mechanisms that would underlie such markers remain unknown.

Accumulating evidence from neuroimaging studies suggests that patients with BD and MDD show reduced brain volume and altered brain metabolism in regions related to emotional and cognitive processing, such as the orbital, inferior, medial, and dorsolateral prefrontal cortex, as well as in subcortical regions involving the hippocampal/amygdala complex and the striatum [2–9]. For instance, meta-analysis studies of voxel-based morphometry (VBM) have revealed that patients with BD exhibit small gray matter (GM) volumes in the inferior frontal/anterior insula [2,3], medial frontal/anterior cingulate cortex (ACC) [2], and temporal regions [3], while patients with MDD show small GM volumes in the ACC [5,6] and dorsolateral and dorsomedial prefrontal cortex [6]. Moreover, a few volumetric neuroimaging studies have directly compared patients with BD and MDD to identify disorder-specific abnormalities. Relative to patients with MDD, patients with BD show reduced GM volumes in the habenula [10], middle cingulate gyrus [11], hippocampus, and amygdala [12], while those with BD have larger GM volumes in the anterior cingulate gyrus [12]. In functional magnetic resonance imaging (fMRI) studies, patients with BD demonstrated altered resting- state brain activity and connectivity relative to patients with MDD[13, 14]. Although these results indicate that patients with BD and MDD have differences in the brain, disorder-specific structural abnormalities remain unclear.

Epidemiological genetic studies of BD and MDD can also be used to evaluate biological markers for distinguishing between the two disorders. Family studies show a relative risk of 10.7 for BD in first-degree relatives (FDRs) of BD probands, while the comparable relative risk for MDD is 2.8 [15]. Moreover, a twin study has shown that the heritability of BD is twice as high as that of MDD [16]. These results suggest a stronger genetic vulnerability for BD than for MDD. Unaffected FDRs of probands are also known to be at higher risk of developing BD or MDD than the general population [17, 18]. Sub-clinical traits of mood disorders show additional common traits that have been defined as candidate endophenotypes of mood disorders among unaffected relatives as compared to the general population [19].

There have been a few VBM studies on FDRs of patients with BD and MDD. For example, it has been shown that the FDRs of individuals with BD exhibit smaller [20] and larger GM volumes [21,22] in the inferior frontal gyrus and insula than healthy control subjects. Moreover, the FDRs of individuals with MDD show reduced hippocampal volumes [23] and increased amygdala volumes as compared to healthy control subjects [24]. Twin studies have also demonstrated decreased hippocampal volumes in FDRs of individuals with BD and MDD, as compared to healthy control subjects [25,26]. These results suggest that brain volumetric abnormalities reflect the vulnerability of FDRs and may reveal candidate endophenotypes for BD and MDD. However, there is, to our knowledge, no study comparing morphometric abnormalities of patients with BD and MDD to their respective FDRs in order to identify disorder-specific volumetric changes.

To facilitate discrimination of BD from MDD, we investigated differences in brain volume and function between patients with BD and the FDRs of individuals with BD (BD group) and patients with MDD and the FDRs of individuals with MDD (MDD group). We examined brain volumes using VBM in both groups, and explored executive functioning in these patients, as impaired executive function has been revealed as a candidate endophenotype of BD [27]. Based on prior VBM studies of individuals with BD and MDD, we hypothesized that BD and MDD groups would show GM volume changes in regions related to emotional and cognitive processing. Such regions would include the medial frontal/ACC and inferior frontal/insula cortex, as well as subcortical regions. We also expected to observe abnormalities in executive function, and that these deficits would be more pronounced in the BD group than in the MDD group.

## Materials and Methods

### Participants

This study was approved by the Institutional Review Board of Yamaguchi University Hospital. Written informed consent was obtained from all participants after a complete description of the study was provided.

Our study consisted of 81 subjects, which included 10 patients with BD and 10 FDRs of individuals with BD (BD group), 17 patients with MDD and 17 FDRs of individuals with MDD (MDD group), and 27 healthy control subjects who had no immediate family members that had any psychiatric disorder (Table 1). Among the patients with BD, seven were diagnosed with bipolar I disorder (BD-I) and three with bipolar II disorder (BD-II). In patients with MDD, seven were undergoing their first major depressive episode, while 10 were classified as having recurrent episodes. All patients were screened by the International Neuropsychiatric Interview (M.I.N.I., Japanese version 5.0.0) [28] and underwent an interview to confirm their diagnosis by psychiatrists according to the DSM-IV-TR. Patients who had a history of electroconvulsive therapy or comorbid psychiatric disorders, including anxiety disorders and current or previous substance use disorders, were excluded.

**Table 1:** Demographics and clinical characteristics of participants.

	HC (n = 27)	BD group (n = 20)		*p*	MDD group (n = 34)		*p*
		patients (n = 10)	FDRs (n = 10)		patients (n = 17)	FDR (n = 17)	
Age (y)	48.3 ± 13.0	46.9 ± 12.3	54.8 ± 20.1	0.409	51.8 ± 11.4	45.5 ± 14.5	0.37
Gender(M/F)	10/17	3/7	5/5	0.109	7/10	5/12	0.03
Premorbid IQ	99.7 ± 7.9	107.4 ± 2.6	97.9 ± 2.6	0.049	94.8 ± 2.0	97.8 ± 2.0	0.097
Years of education (y)	14.0± 2.0	14.7± 1.6	12.7± 2.5	0.094	13.8± 2.3	13.4± 2.0	0.64
Handedness	88.7± 12.7	91.0± 13.9	97.0± 5.4	0.179	90.0± 15.8	86.5± 24.5	0.84
GAF	99.4± 2.1	42.0± 17.0	98.3± 3.3	< 0.01	40.3± 7.8	97.5± 3.1	< 0.01
HDRS	0.3± 0.7	21.3± 6.7	0.2 ± 0.4	< 0.01	23.5 ± 5.1	0.4± 1.0	< 0.01
YMRS	0.0 ± 0.0	0.0 ± 0.0	0.1± 0.3	0.158	0.2 ± 0.5	0.06± 0.1	0.18
Antipsychotic doses		181.4± 113.7			81.0± 69.8		0.072
Antidepressants doses		158.8± 36.1			211.7± 96.2		0.25
Duration of illness (m)		74.1± 110.3			49.5± 65.1		0.8
Onset age of illness (y)		32.2 ± 11.5			43.6 ± 13.9		0.047
The number of depressive episodes		6.4±5.2			2.2±1.1		< 0.01

HC, healthy control subjects; BD, bipolar disorder; MDD, major depressive disorder; GAF, Global Assessment for Function by DSM-IV-TR; HDRS, Hamilton Rating Scale for Depression; YMRS, Young Mania Rating Scale; TMT, Trail Making Test; the value was represented the mean ± SD. Statistics were done by ANOVA or Mann-Whitney test.

Current mood states of the patients were evaluated by a 17-item Hamilton Depression Rating Scale (HDRS) [29] and the Young Mania Rating Scale (YMRS) [30]. History of suicide was rated in six grades: 1, “never”; 2, “a brief thought”; 3, “planned at least once but did not attempt”; 4, “planned at least once and really wanted to die”; 5, “attempted suicide but did not want to die”; 6, “attempted suicide and hoped to die”. All patients were taking mood stabilizers (10 with BD, one with MDD), antidepressants (four with BD, 17 with MDD), or antipsychotics (second-generation antipsychotics: seven with BD, four with MDD; first-generation antipsychotics: two with BD, three with MDD). Eight of the 10 patients with BD took lithium. Antipsychotic doses were estimated using chlorpromazine dose equivalents and antidepressant doses were estimated using imipramine dose equivalents.

Of the FDRs of individuals with BD, four had a parent with BD, four had a sibling with BD, and two had a child with BD. In FDRs of individuals with MDD, three had a parent with MDD, 12 had a sibling with MDD, and two had a child with MDD. The FDRs were screened by the M.I.N.I. and were excluded from the study when the examination revealed any psychiatric disorders. Twenty-seven healthy control subjects were screened using the M.I.N.I., and were excluded if any of their immediate family members had psychiatric disorders or heritable neurological diseases.

Exclusion criteria for all participants included the presence of neurological or endocrine diseases, or abnormal results on medical laboratory tests, such as hypothyroid function, head trauma, Parkinson’s disease, family history of hereditary neurological disorder, severe hypertension and diabetes, active liver disease, kidney problems, and respiratory problems. All subjects were right-handed, as assessed using the Edinburgh handedness inventory [31]. The Global Assessment of Function (GAF) from the DSM-IV-TR was used to assess social functioning. Premorbid IQ scores were estimated using the Japanese Adult Reading Inventory (JART) [32].

### Magnetic resonance imaging

Brain images were collected on a 1.5-tesla Magnetom Vision scanner (Siemens Medical System Inc., Erlangen, Germany). A three-dimensional gradient-echo sequence (FLASH, fast low-angle shot) yielding 160–180 contiguous slices (1.0 mm thick) in the sagittal plane was used for image analysis. The MRI parameters were as follows: echo time = 5 ms, repetition time = 24 ms, flip angle = 40°, field of view = 256 mm, matrix size = 256 × 256, voxel size = 1 × 1 × 1 mm. MR images were manually checked for quality before the VBM analysis, and abnormal findings were identified by psychiatrists with expertise in MRI studies, as well as by radiologists who were blinded to the subjects’ diagnosis.

### Image analysis

Image preprocessing was performed using SPM8 software (Wellcome Department of Imaging Neuroscience, London, UK) using Matlab R2012b 8.0.0.783 (MathWorks, Natick, MA, USA). T1-weighted images were segmented and imported into a format that could be used by the VBM8 algorithm. The segmented images were normalized to the Montreal Neurological Institute (MNI) space and smoothed with an 8-mm Gaussian filter.

### Executive function

Cognitive function was assessed by a trail making test (TMT). Recent meta-analyses of executive function in individuals with BD and MDD have shown poorer performance in patients than in healthy control subjects [33, 34]; thus, poor executive function could be a candidate endophenotype for BD [35]. Here, we used the time it took to complete a task for assessing function. The test consists of two parts (A and B) that must be performed as quickly as possible. Part A required individuals to connect a series of 25 numbered dots randomly distributed on a sheet of paper by drawing a line between them (1-2-3…), and assessed visual attention and processing speed [36]. Part B required individuals to connect a series of alternating numbered and lettered dots in order (1-A-2-B…), and assessed set-shifting [37]. The difference between the time it took subjects to complete Part A and Part B (TMT B-A) was also determined. The TMT B-A score is meant to remove the speed component from the test evaluation and represents executive function; it has been reported as a sensitive tool for assessing frontal lobe function [38]. Higher scores on the TMT B-A represented lower executive function. Furthermore, the score of the TMT was reportedly associated with medial frontal, insula, and ACC volumes [39, 40].

### Statistical analysis

#### VBM

We used SPM8 software to implement a general linear model analysis. First, we performed a whole-brain analysis using a factorial design, with age, sex, and premorbid IQ scores as covariates, in SPM8, to determine whether there was an effect of diagnosis when comparing either the BD or the MDD group with healthy control subjects. A voxel-wise *F*-test was performed with the threshold set at *p* < 0.05, corrected by a family-wise error (*p*_*FWE-corr*_). Mean GM volumes of regions that showed a significant main effect of diagnosis were then extracted by MarsBar (http://marsbar.sourceforge.net/) and compared among patients, FDRs, and healthy control subjects using an analysis of variance (ANOVA) in SPSS for Windows statistical software, version 16.0 (SPSS, Inc., Chicago, IL). We anatomically identified regions using automated anatomical labeling via WFU PickAtlas version 2.4 (http://www.fmri.wfubmc.edu/download.html).

Second, another whole brain analysis was carried out in SPM8 using a factorial design with the condition having two factors (patients vs. FDRs) and diagnosis having two factors (BD vs. MDD) to determine whether there was an interaction between condition and diagnosis.

#### TMT

We used the Kruskal–Wallis test for TMT Parts A, B, and B-A among the five groups. The Mann–Whitney U test was also used to compare differences between the two groups. Statistical significance was set at p < 0.05.

#### Medication effect

To examine the effects of medication load on brain volume, we calculated the dosage of psychiatric medications. Antipsychotic doses were estimated using chlorpromazine dose equivalents. Antidepressant doses were estimated using imipramine dose equivalents. Mood-stabilizer medication was coded as absent (0), taking lithium (1), or taking lithium and valproate (2).

#### Correlation analysis

A correlation analysis was applied to determine the relationship between significant results on the VBM and TMT. We also performed correlation analysis of the following clinical variables: age of illness onset; duration of illness; numbers of depressed, manic, and total episodes; scores on the HDRS and YMRS; medication load, and suicide history.

## Results

### Demographics

There was no significant difference in mean age, premorbid IQ, handedness, and years of education among the patients, FDRs, and healthy control subjects (Table 1). The distribution of sex among patients with MDD, FDRs of individuals with MDD, and healthy control subjects was significantly different. The mean age of onset in patients with BD was earlier than that of patients with MDD (*U* = 45.5, *p* = 0.05 by Mann–Whitney *U* test). No significant difference was observed between patients with BD and MDD in terms of illness duration (*U* = 80.0, *p* = 0.82) or in use of antipsychotics and antidepressants (*U* = 12.5, *p* = 0.072; *U* = 22.0, *p* = 0.25, respectively). Patients with BD had more mood episodes than patients with MDD (*U* = 9.0, *p* < 0.001). Eight of 10 patients with BD took lithium.

### VBM

**Fig 1:**
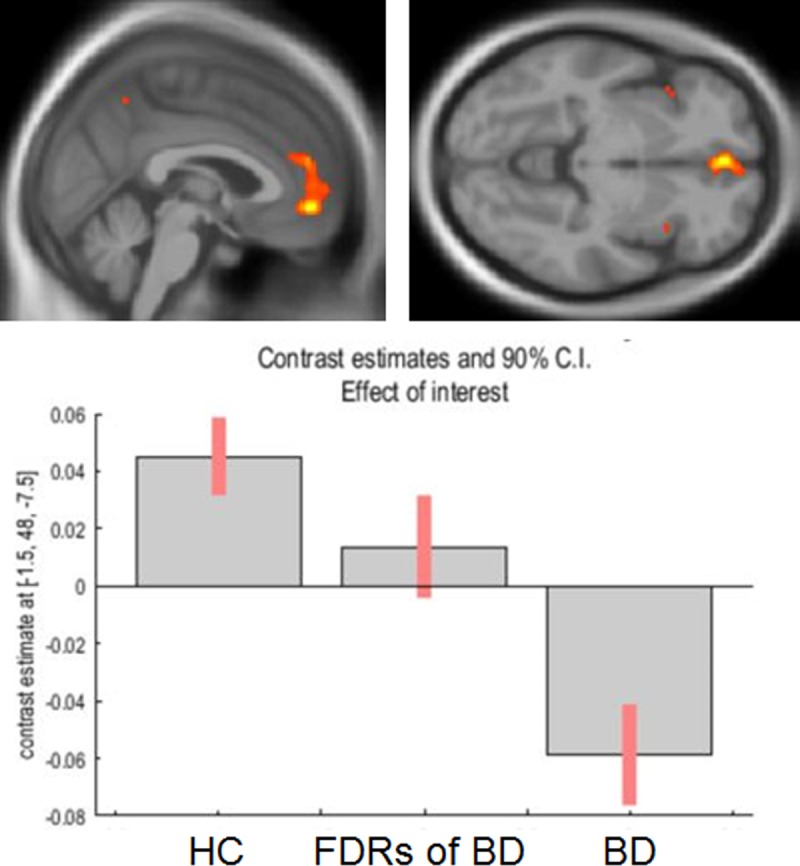
Anterior cingulate GM volumes among the BD group in the whole brain analysis. The patients with BD and FDRs of individuals with BD showed significantly smaller volumes compared to healthy control subjects. FDR first-degree relatives; HC healthy subjects.

**Fig 2:**
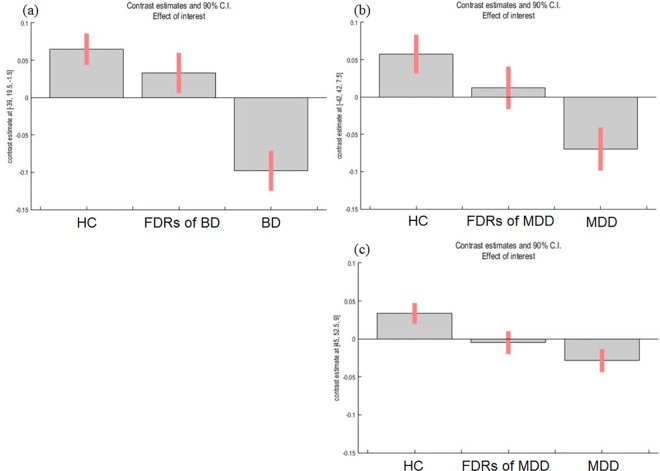
Comparisons of GM volumes in the left and right insula of patients in BD and MDD groups. Patients with BD showed significantly smaller GM volumes in the left (a) and insular cortex compared to healthy control subjects. There was a trend of a main effect of diagnosis in the GM volume of the left (b) and right (c) insular cortex in the MDD group.

For the BD group, a significant main effect of condition was found in the GM volume of the left medial prefrontal cortex adjacent to the ACC (coordinates of the voxel of maximum statistical significance: x = -1.5, y = 48, z = -7.5, F = 22.96, k = 13, *p*_*FWE-corr*_ = 0.01; Fig 1) and left insula (*x* = -39, *y* = 19.5, *z* = -1.5, *F* = 29.34, *k* = 103, *p*_*FWE-corr*_ < 0.01; Fig 2(A)) in the whole brain analysis. In a *post hoc* analysis, patients with BD and FDRs of individuals with BD showed significantly smaller GM volumes in the left ACC than healthy control subjects (*p* < 0.01, 95% confidence interval, 95%CI [0.03, 0.13]; *p* < .05, 95% CI [0.002, 0.11], respectively). Moreover, patients with BD showed significantly smaller GM volumes in the left insula than did healthy control subjects, *p* < .01, 95% CI [0.05, 0.21]. There was no significant correlation between any medication load and left ACC or left insular GM volume in patients with BD (antipsychotics; r = -0.66, p = 0.076; r = -0.36, *p* = 0.93, respectively, antidepressants; r = -0.32, *p* = 0.68; r = -0.32, *p* = 0.68, respectively, and mood stabilizers; r = 0.23, p = 0.52; r = 0.23, *p* = 0.52, respectively).

**Fig 3:**
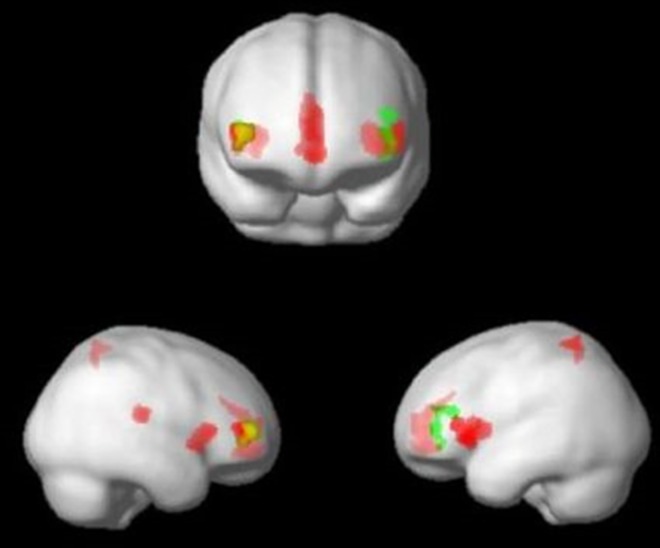
A superimposed image of results from the whole-brain analysis. Areas in red represent regional GM volumes that showed a significant main effect of diagnosis in the BD group. Areas in green represent regional GM volumes that showed a main effect of diagnosis in the MDD group. Areas in yellow represent shared results of a main effect of diagnosis in BD and MDD groups.

For the MDD group, there was a significant main effect of condition in the GM volume of the left and right inferior frontal gyrus adjacent to the insula (*x* = -42, *y* = 42, *z* = 7.5, *F* = 12.53, *k* = 347, uncorrected *p* = .05 × 10^−4^; *x* = 45, *y* = 52.5, *z* = 9, *F* = 14.18, *k* = 248, uncorrected *p* < .01 × 10^−4^, respectively) in the whole brain analysis (Fig 2(B) and 2(C)), although neither of these were significant after FWE correction. We overlaid the results of the whole-brain analysis of the BD group with those of the MDD group and found that the ACC was a region that was identified specifically in the BD group, while the insula was a shared region that was highlighted in both groups (Fig 3).

A two-by-two ANOVA of the whole brain revealed that there was no significant main effect of condition (patients vs. FDRs) or diagnosis (BD vs. MDD). Furthermore, there was no interaction between condition and diagnosis.

### TMT

Patients with BD took a significantly longer time to complete Part B, and had higher scores for the B-A of the TMT than did healthy control subjects (U = 76.0, *p* = 0.044; U = 66.0, *p* = 0.017, respectively). Patients with MDD took a significantly longer time to complete Part A and Part B than did the FDRs of individuals with MDD (U = 12.5, *p* < 0.001; U = 53.5, p = 0.001, respectively) and healthy control subjects (U = 91.0, *p* = 0.001; U = 80.0, *p* < 0.001, respectively) (Table 2).

**Table 2:** The results of behavioral performance and statistics in the score of TMT.

TMT scale	Behavioral performance (sec), *M (SD)*					*p*
	BD group		MDD group		HC	

	patients	FDRs	patients	FDRs		
A	55.2 (43.4)	35.3 (14.6)	49.8 (13.2)	27.5 (5.9)	33.4 (13.7)	< 0.01
B	126.5 (90.0)	98.1 (65.3)	126.1 (78.8)	67.4 (25.9)	67.4 (24.3)	< 0.01
B-A	71.3 (57.4)	62.8 (51.1)	76.3 (72.4)	39.9 (23.5)	34.0 (16.7)	0.12

BD Bipolar disorder; MDD Major depressive disorder; FDR First degree relatives; HC Healthy control subjects.

### Correlations

**Fig 4:**
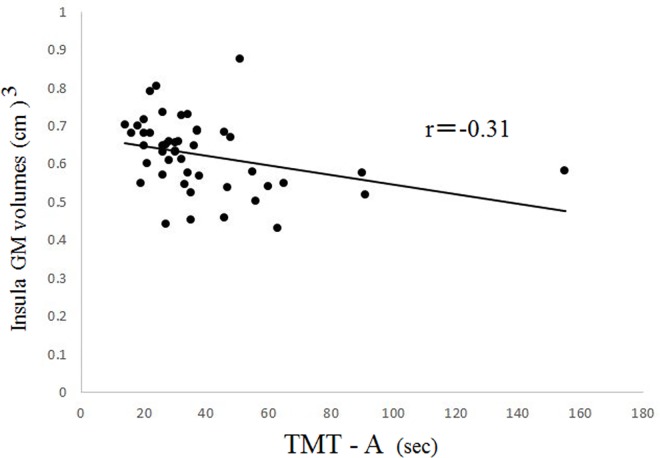
The correlation between GM volumes of the left insula and the time it took patients to complete Part A of the TMT. The left insular GM volume was negatively correlated with scores of the TMT-A in the BD group.

The BD group showed a significantly inverse correlation between GM volumes of the left insula and the time it took patients to complete Part A of the TMT (*r* = -0.31, *p* < 0.05 by Spearman's rho test; Fig 4). When excluding the subject with a TMT score of 160, who appeared to be an outlier, the result was still significant (r = -0.46, *p* < 0.05).

In patients with BD, the severity of suicide history was also inversely correlated with GM volumes of the left insula (*r* = -0.76, *p* = 0.011 by Spearman's rho test). No other significant correlations were found between VBM and TMT data and any clinical variables in the BD or MDD groups.

## Discussion

Our current study revealed a reduction in the GM volume in the ACC of patients with BD and FDRs of individuals with BD, but not in patients with MDD or in FDRs of individuals with MDD. A whole brain analysis revealed a significant main effect of condition in the GM volume of the insula in patients with BD and MDD, as well as a reduced insular GM volume in patients with BD. We further found delayed TMT performance in patients with BD and MDD, but not in the FDRs of individuals with BD or MDD. Finally, we found that the smaller insular GM volumes were associated with poor performance on the TMT-A as well as a severe suicide history in patients with BD. These findings suggest that the ACC GM volume could represent a distinct endophenotype of BD and that the insular GM volume could be a shared endophenotype of BD and MDD. Further, these findings suggest that insular GM volume may indicate cognitive decline and poor outcome in individuals with BD, and moreover, that performance on the TMT cannot distinguish between BD and MDD.

The ACC plays a key role in the regulation of emotion and executive function [41], and disruption to this region is involved in the pathophysiology of BD. Some meta-analytical studies have supported the evidence that patients with BD have small GM volumes in the ACC [2, 42–44]; however, other studies have not reported this reduction [3, 45–47]. For example, previous VBM studies on relatives of individuals with BD have shown that genetic risk for BD is associated with a reduced ACC GM volume [48]. Moreover, it has been shown that FDRs of individuals with BD have smaller medial white matter volumes near the ACC than do healthy subjects [20]. In contrast, a manual-tracing study showed that unaffected offspring of parents with BD do not have statistically significantly different subgenual ACC volumes when compared to healthy control subjects [49]. The negative findings of the latter study may be due to the use of different imaging analyses from the former two studies. Moreover, the latter study tested younger participants (mean age, 19−20 y); thus, the unaffected offspring of individuals with BD may potentially include individuals with BD before onset. Our findings support the evidence that the ACC volume may be a candidate endophenotype of BD and that the ACC may be involved in the morphometric pathophysiology related to the genetic liability for BD.

Another interesting finding of this study was that individuals in the BD and MDD groups exhibited decreased insular GM volumes. The insular cortex plays a crucial role in emotional arousal and feeling [50, 51], and is assumed to be involved in a network model of higher-level cognitive control and emotional processing [52]. Three previous meta-analyses of VBM findings have revealed that patients with BD show smaller insular GM volumes than do healthy control subjects [2,3,53]. Two family studies of BD have also supported evidence of a change in insular volume, with one study on patients with BD and FDRs of individuals with BD showing a smaller left anterior insular GM volume than seen in healthy control subjects [20] and the other study showing an association between increased volume of the left insula and a genetic predisposition to BD [23]. Although more family studies of BD are required to confirm whether increases or decreases in GM volume are indicative of BD predisposition, the findings of the present study, together with those of prior studies, indicate that insular GM volume is associated with the genetic liability for BD.

The present study also demonstrated that insular GM volume was associated with a severe history of suicide in patients with BD, as well as scores for the TMT-A in patients with BD and FDRs of individuals with BD. In previous studies, it has been reported that individuals with BD that have attempted suicide have smaller insular GM volumes than do individuals with BD that have not attempted suicide [54] and patients with BD with a history of psychotic episodes and psychotic spectrum disorders [55]. It has also been reported that patients with depression and a history of attempted suicide take a significantly longer time to complete the TMT-A than do healthy control subjects, whereas no significant differences have been found between the time it takes patients with depression without a history of attempted suicide to complete the TMT-A and healthy control subjects [56]. The results of the present study therefore further support the hypothesis that the insula may play a role in mediating suicidal ideation and the execution of suicidal behavior in individuals with BD. Previous reports have indicated an association between the insular cortex and TMT performance in healthy control subjects [57, 58]. In addition, the insula is reported to be part of a frontal–striatal attention network [59]. These finding suggest that the insula may be involved in executive function as measured on the TMT.

In terms of patients in the MDD group, we also found smaller GM volumes in the insula, but this finding was not statistically significant after correction for multiple comparisons. The evidence of the relationship between the insular volume and MDD has been controversial. Although some VBM and manual-tracing studies have reported reduced insular volumes in patients with MDD as compared to healthy control subjects [60,61], meta-analytical volumetric studies have failed to show reductions in this region in patients with MDD [6,62,63]. Furthermore, previous VBM family studies have not shown significant differences in the insular GM volume among the FDRs of individuals with MDD, patients with MDD, and healthy subjects [25,64]. The limited evidence of changes in insular volume in individuals with MDD may be partly due to the low genetic liability for MDD as compared to that for BD [15,16]. More familial neuroimaging studies of MDD across larger samples are required to evaluate the association between the genetic liability for MD and brain volume. Nonetheless, the results of the current study suggest that reduced insular GM volume may be involved in the shared pathophysiology of BD and MDD, and moreover, that this is associated with cognitive dysfunction and the grave outcome of individuals with BD.

In terms of executive function on the TMT, we found that patients with BD and MDD, but not FDRs of individuals with BD or MDD, showed significantly poorer results than did healthy control subjects. Previous reports have demonstrated that patients with BD and MDD exhibit cognitive impairments on the TMT-A [65–67] and TMT-B [65,66,68,69] as compared to healthy control subjects. These findings are consistent with our results. However, our findings on the cognitive function of the FDRs of individuals with BD and MDD are inconsistent with several previous reports. For instance, it has been reported that the FDRs of individuals with BD show deficits in response inhibition but not in working memory or cognitive set-shifting as compared to healthy control subjects [70]. Another study has reported that the FDRs of individuals with BD show impairments in verbal working memory, but not in psychomotor performance as compared to healthy control subjects [71]. Additionally, a meta-analysis on neuropsychological tests has reported that cognitive impairments in response inhibition, set-shifting, and sustained attention are observed in patients with BD and the FDRs of individuals with BD, while processing speed, verbal working memory, and visual memory are affected in patients with BD, but not in the FDRs of individuals with BD [72]. These results suggest that, although impaired executive functioning may be one promising endophenotype of mood disorders, deficits in the FDRs of individuals with mood disorders may be restricted to a certain domain of executive function.

The current study had several limitations. Fist, the small sample size may have limited our statistical power. Second, all patients with BD and MDD that participated in the study were taking psychiatric medications. Some reports have demonstrated that antidepressant administration increases the GM volume of the dorsolateral prefrontal cortex [73] and hippocampus [74,75] in patients with MDD, and that lithium impacts cortical GM density [76]. One review has suggested that medicated patients with BD show no significant effects of psychotropic medications in terms of structural and functional neuroimaging measures [77]. Due to this finding, and despite the lack of significant correlations between medication load and volumetric changes in the patients with BD in the present study, we cannot exclude the possibility that medication may have masked our results.

In conclusion, this is the first study to compare candidate endophenotypes related to changes in brain volume between BD and MDD. Our results suggest that the volumetric changes in the ACC represent a distinct endophenotype of BD and that the changes in insular volume constitute a shared endophenotype between BD and MDD. Furthermore, changes in these regions may be associated with the prognosis of BD, indicating that these findings may be used as biomarkers for distinguishing between BD and MDD. Although there were no differences on performance of the TMT between the FDRs of individuals with mood disorders and healthy control subjects, GM volumes in the left insula were significantly inversely correlated with TMT-A scores in patients with BD and FDRs of patients with BD, but not in MDD patients and FDRs of patients with MDD. Future studies should evaluate both volumetric and cognitive changes in order to reveal characteristics specific to BD. For instance, Almeida et al. have indicated that combined dimensional approaches, including neuroimaging and cognitive function studies, may be useful to identify individuals at future risk for BD versus MDD [78]. Thus, further studies are required to identify biomarkers that can be used for the early diagnosis of BD.
